# Preliminary study on the molecular features of mutation in multiple primary oral cancer by whole exome sequencing

**DOI:** 10.3389/fonc.2022.971546

**Published:** 2022-10-20

**Authors:** Kan Li, Jianbin Gong, Qiuhan Zheng, Le Yang, Xueying Mei, Jianghai Chen, Guiqing Liao, Yujie Liang

**Affiliations:** ^1^ Hospital of Stomatology, Sun Yat-sen University, Guangzhou, China; ^2^ Guangdong Provincial Key Laboratory of Stomatology, Sun Yat-sen University, Guangzhou, China; ^3^ Guanghua School of Stomatology, Sun Yat-sen University, Guangzhou, China

**Keywords:** multiple primary oral cancer, whole exon sequencing, mutational signatures, copy number variation, germline mutation

## Abstract

Multiple primary cancers (MPCs) refer to cancers that occur simultaneously or metachronously in the same individual. The incidence of MPC has increased recently, as the survival time of malignant tumor patients has been greatly prolonged. It is difficult to differentiate MPC from primary cancers (PCs) in the same anatomical region from the clinical manifestation alone. However, their biological behaviors appear to be distinct. In this study, we show that the prognosis of multiple primary oral cancers (MP-OCs) is worse than primary oral cancers (P-OCs). To better understand the molecular mechanisms of MP-OC, we used whole exome sequencing (WES) to analyze samples from 9 patients with MP-OC and 21 patients with P-OC. We found more somatic mutations in MP-OC than in P-OC. MP-OC had more complicated mutation signatures, which were associated with age-related and Apolipoprotein B mRNA Editing Catalytic Polypeptide-like (APOBEC) activity-related signatures. Tumor mutational burden (TMB) and mutant-allele tumor heterogeneity (MATH) of MP-OC trended higher compared to P-OC. KEGG and GO analysis showed the differential pathways of MP-OC versus P-OC. In addition, MP-OC took amplification, not loss, as the main pattern of copy number variation (CNV), while P-OC took both. Lastly, we did not find significantly different mutant germline genes, but MSH-6 mutation may be a potential MP-OC driver. In short, our preliminary results show that MP-OC and P-OC have different molecular characteristics.

## 1 Introduction

Multiple primary cancers (MPCs) are cancers that occur simultaneously or metachronously in the same individual. Metastasis of initial primary cancers should be carefully excluded (1). As early as 1889, Billroth first reported a case of multiple primary gastric cancer after epithelial carcinoma of the outer ear (2). In 1932, Warren and Gates formally determined the diagnostic criteria of multiple primary cancers (3). MPC was once regarded as a rare disease (4). With the improvement of early screening, diagnostic techniques, treatment methods, postoperative follow-up, and supportive treatment, survival following malignant tumors has been greatly prolonged. Meanwhile, the incidence rate of MPC has significantly increased (5–7). Without a clear medical history, there are few obvious differences between MPC and primary cancers (PCs) in terms of their clinical manifestations. However, Kai-Hsiung et al. reported that the 5-year overall survival and 5-year disease-free survival were worse in individuals with multiple primary lung cancers than those with a primary lung cancer (8). Qi-Wen et al. showed that compared with matched cases, esophageal squamous cell carcinoma accompanied with synchronous MPC was related to significantly impaired survival and an elevated risk of locoregional disease progression following concurrent chemoradiotherapy (9). It thus appears that the underlying biological behavior of these two clinical groups might be distinct. A better understanding of the clinical features and molecular mechanisms of multiple primary cancers may be beneficial to cancer management. However, current studies on multiple primary cancers had mostly focused on its clinicopathological features (10). There are only a handful of studies that reveal the differences in the molecular mechanisms of MPC.

Gene mutations, including single-nucleotide polymorphism (SNP), insertion–deletion (InDel), and copy number variation (CNV), play an important role in the occurrence and progression of tumors. For example, the *RB1* gene mutation results in retinoblastoma (11). Detection of the mutation helps to reveal the molecular mechanisms of MPCs and to promote prognostication and treatment planning. Whole exome sequencing (WES) is the most common method used to detect gene mutations. Tumor mutational burden (TMB) and microsatellite instability (MSI), which have been approved as pan-cancer biomarkers by the FDA, can also be obtained by WES (12, 13). Patients with high TMB (TMB-H) or high MSI (MSI-H) benefit from immunotherapy.

In this study, we found that the prognosis of individuals with multiple primary oral cancers (MP-OCs) was worse than those with primary oral cancers (P-OCs) (8). We used WES to analyze 9 patients with MP-OC and 21 patients with P-OC, to better understand the underlying molecular mechanisms. The mutant germline genes of two groups were also analyzed.

## 2 Materials and methods

### 2.1 Dataset and bioinformatics analysis

The SEER database is currently the largest publicly available cancer database, covering approximately 28% of the US population (14). We chose SEER Research Data, 9 Registries, Nov 2020 Sub (1975–2018) to calculate the proportion of MP-OC in oral cancer (OC). Then, we analyzed the survival of MP-OC and P-OC. SEER*Stat software (version 8.3.92; National Cancer Institute, Bethesda, MD, USA) was used to analyze the proportion of MP-OC from 1975 to 2018.

The range of OCs included tongue, floor of mouth, and gum and other mouth. According to the International Classification of Diseases for Oncology, third edition (ICD-O-3), the codes for tongue cancer are C019–C029, those for floor of mouth cancer are C040–C049, and those for gum and other mouth cancers are C030–C039, C050–C059, and C060–C069.

Data on sex, site of cancer, histopathological type, survival time, and cause of death were collected. OC patients with a history of malignancies were classified as MP-OC, and the remaining cases were classified as P-OC. We excluded patients with ambiguous information on survival, which was labeled with “N/A not seq 0-59”.

### 2.2 Patient cohort

A total of 317 patients with OC from the Hospital of Stomatology of Sun Yat-sen University (Guangzhou, China) were enrolled from October 2014 to September 2016 to further confirm the different prognoses of MP-OC and P-OC. Of these, 16 were MP-OC patients. The inclusion criteria of MP-OC followed the SEER criteria (15): multiple neoplasms were malignant with a definite pathological diagnosis; multiple cancers come from different tissue origins, or the edges of the cancer lesions are at least 2 cm apart from each other; if multiple cancers were of the same site and tissue origin, their times of diagnosis had to be at least 5 years apart. Recurrence and metastasis were carefully excluded. The endpoint of follow-up was 31 December 2020.

A total of 64 patients were enrolled for WES and subsequent analyses, including 20 with MP-OC and 44 with P-OC. Fresh cancerous tissues were collected from 30 patients for somatic genome mutation analysis, including 9 with MP-OC and 21 with P-OC. Blood or normal mucosa was taken as controls. We also collected blood or mucosal samples from all remaining participants. These samples were sequenced for germline gene mutations. The samples were stored in liquid nitrogen or at −80°C until WES. The DNA libraries were sequenced on the Illumina sequencing platform by Gene Denovo Honour Biotechnology Co., Ltd (Guangzhou, China).

### 2.3 Detection and analysis of somatic SNV and InDel

#### 2.3.1 Nucleic acid preparation

DNA was extracted using the Cetyltrimethyl Ammonium Bromide (CTAB) method to generate libraries for Next-Generation sequencing. NEBNext dsDNA Fragmentase (NEB, Ipswich, MA, USA) was used to fragment genomic DNA ends followed by DNA end repair. Then, NEBNext adaptor (NEB, Ipswich, MA, USA) was used to dA-tail and ligate these DNA fragments. Biotinylated RNA library baits and magnetic beads were mixed with the barcoded library to select targeted regions with the SureSelect Human All Exome V6 Kit (Agilent Technologies, Palo Alto, Calif.). Next, we further amplified the captured sequences for 150-bp paired-end sequencing using the Illumina X-ten system (Illumina, San Diego, CA, USA).

#### 2.3.2 Clean reads filtering

Fastq (16) was used to filter the raw reads. The low-quality parts of raw data were identified for removal as follows: reads with ≥10% unidentified nucleotides; reads with > 50% bases having phred quality scores of ≤20; and reads aligned to the barcode adapter. The quality of clean data after filtering is shown in **Supplementary Figures S1A, B**.

#### 2.3.3 Somatic SNV and InDel identification

The Burrows–Wheeler Aligner (BWA) (17) completed the alignment of the clean reads against the human reference genome (GRCh38). Somatic SNV and InDel of multisample were called by MuTect (18).

#### 2.3.4 Analysis of mutational signatures

The non-negative matrix factorization (NMF) method was used to obtain mutational signatures (19). There were 96 possible mutation types combined to obtain a matrix representing each feature. By cosine similarity, the identified signatures were compared to the previously determined consensus signatures (20) and confirmed.

#### 2.3.5 Identification of mutated genes

Considering all variations, including somatic SNV and InDel, we identified the set of mutated genes. Genes with a *p*-value<0.05 were considered to be with high-frequency mutations. Significantly mutated genes (SMGs) refer to the genes whose mutation frequency was significantly higher in cancer than in controls. We used MuSiC (21) to search for SMGs. MuSiC is a method based on the mutational recurrence on all cancer samples. A convolution test was carried out on each mutation type. Only the mutated genes with FDR<0.01 were considered to be SMGs.

#### 2.3.6 Gene functional enrichment analysis

The set of mutated genes with a *p* value<0.05 were used for gene functional enrichment analysis. The Kyoto Encyclopedia of Genes and Genomes (KEGG) (22) and Gene Ontology (GO) (23) are commonly used databases. We used an in-house script that incorporated both databases for functional annotation analysis. The significance of gene group enrichment was defined by a modified Fisher’s exact test and *Q* value< 0.05 was considered to indicate a statistically significant difference.

### 2.4 Calculation of TMB, MSI, and MATH

Nonsynonymous mutations in the coding region were selected to assess TMB. We used 38 Mb as the estimate of the exome size. All nonsynonymous mutations/exome lengths indicate the TMB of each sample (24).

Microsatellites are repetitive sequences with unit lengths varying from 1 to 6 bp. We evaluated the MSI following the MSIsensor method described by Niu and Ding (25).

Mutant-allele tumor heterogeneity (MATH) is a quantitative assessment of genetic intra-tumor heterogeneity, based on WES of tumors and matched normal tissues. The MATH score was calculated following the method described by Mroz and Rocco (26). Briefly, the MATH value of each tumor was calculated from the median absolute deviation (MAD) and the median of its mutant-allele fractions at tumor-specific mutated loci using:


MATH=100∗MAD/median.

### 2.5 Somatic CNV detection and analysis

VarScan 2 (27) was used to identify somatic CNVs with the following parameters: minimum coverage ≥20 and phred base quality ≥20. DNA copy packages in R/Bioconductor were used to normalize the log ratio of coverage between tumor and normal samples by circular binary segmentation. Recurrent somatic CNVs were identified by using GISTIC (28). GISTIC broad-level analysis was performed with a size threshold of 98% of a chromosome arm to differentiate between focal events and arm level. *q* ≤ 0.25 was considered significant for arm-level events and CNV regions. Then, residual *q*-values *q* ≤ 0.05 were used to determine the significance of focal CNV events. All oncogenes and tumor suppressor genes were identified according to the OncoKB™(https://www.oncokb.org/cancerGenes).

### 2.6 Detection and analysis of germline mutant genes

According to the study of Kuan-Lin Huang et al. (29), 152 genes were identified as cancer predisposition genes (**Supplementary Table S1**). We annotated and prioritized pathogenic variants in these 152 genes in our 64 patients.

The tools used were as follows: germline SNPs were identified using VarScan2 (30) (version 2.3.9) and GATK (31) (version 3.81); germline InDels were identified using VarScan2, GATK, and Pindel (32) (version 0.2.5b9); and pathogenicity was assessed using SIFT (33) and PolyPhen (34).

We set the filter conditions as follows: firstly, only variants with a structure type of exonic or splitting were retained; we made requirements for allelic depth (AD) and read depth (RD), requiring AD ≥5 and AD/(RD + AD) ≥0.2; we then removed variants potentially contaminated by tumor-adjacent tissues. Variants were removed if they were found in both cancer and tumor-adjacent tissues, and the allele fraction in tumor-adjacent tissues was<0.3; finally, we collected all SNPs called by GATK and VarScan2, and indels that were called by at least two of the three callers (GATK, VarScan2, and Pindel).

We used several methods to score these variants, and finally obtained the sum of the scores of the variants. A higher score indicated higher pathogenicity. A final score of ≥1 was classified as pathogenic, while variants with scores of< 1 were discarded. The specific methods are as follows:

Firstly, we required variants with a dominant mode of inheritance; variants would be removed if they did not meet this requirement.

Secondly, the pathogenicity of polypeptide changes was annotated in ClinVar; variants were given +7 points if consistent with previously identified pathogenic variants in ClinVar. Variants that resulted in a different amino-acid change at the same position were given +2 points.

Thirdly, we used HotSpot3D (35) to analyze the protein structure and pathogenicity; if a germline variant was found to be a recurrent somatic mutation among all cancer types in a HotSpot3D cluster, the variant was given +2 points.

Fourthly, we scored according to mutation frequency. Variants that did not exist or showed a very low frequency (MAF< 0.0005) in the ExAC dataset were given +2 points; common variants (MAF > 0.05) were given −8 points.

Finally, we used SIFT and PolyPhen for scoring. If a variant was identified as “damaging” or “deleterious” in SIFT (score<0.05) and “probably damaging” in PolyPhen (score >0.432), it was given +1 point. Conversely, if SIFT is greater than 0.05 and PolyPhen is less than 0.432, the 1 point would be taken away.

### 2.7 Statistical methods

Statistical analysis was carried out by using IBM SPSS Statistics 25.0 (IBM Corp., Armonk, NY, USA). Clinical data including age, gender, site of OC, smoking history, and excessive drinking history were compared between P-OC and MP-OC using the Mann–Whitney *U* test, Chi-square test, or Fisher’s exact test. The difference of sequencing results between two groups such as TMB, MATH, and MSI was also analyzed by the Mann–Whitney *U* test, Chi-square test, or Fisher’s exact test. The survival rate of MP-OC and P-OC was calculated by using the Kaplan–Meier method, and the survival curve was generated. We further performed univariate regression analyses and a multivariate Cox regression analysis. Candidates were subjected to a multivariate Cox proportional hazards regression with the enter selection of variables (*α* = 0.1), and the forest plot was drawn to show the hazard ratio of risk factors. The log-rank test was used to formally test the differences. Significance was determined based on a *p*< 0.05 threshold.

## 3 Results

### 3.1 The proportion of MP-OC among oral cancers has increased over time

**Figure 1:**
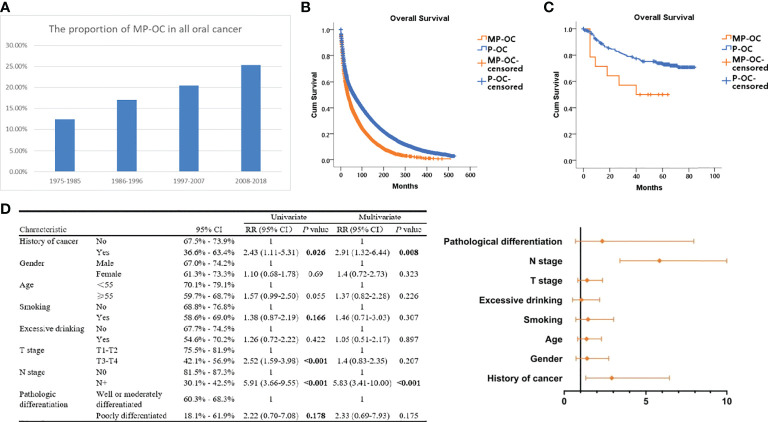
Proportion and prognosis of MP-OC and P-OC patients. Histogram of MP-OC patients’ proportion with years **(A)**. Overall survival of MP-OC and P-OC patients from the SEER database. Kaplan–Meier survival analysis, *p*< 0.001 **(B)**. Overall survival of matching 16 MP-OC patients and 301 P-OC patients from the Hospital of Stomatology, Sun Yat-sen University. Kaplan–Meier survival analysis, *p* = 0.03 **(C)**. Univariate regression analyses and multivariate Cox regression analysis of OC patients **(D)**.

Data from SEER showed that the proportion of MP-OC among OCs increased over time. Among all OC patients between 1975 and 1985, MP-OC patients accounted for 12.73%, while the proportion increased to 25.32% between 2008 and 2018 (**Figure 1A**).

### 3.2 MP-OC shows worse prognosis than P-OC

We first investigated the patients in the SEER database. The survival of patients with MP-OC was significantly worse, with a median survival time of 70.52 months (95% CI, 68.52–72.52), compared with 119.02 months (95% CI, 117.50–120.53) in the P-OC group (Kaplan–Meier survival analysis, *p<* 0.001) (**Figure 1B**). To confirm this result, we enrolled 317 patients with OC from our center; their clinicopathologic features are listed in **Table 1** (further details are provided in **Supplementary Table S2**). There was no significant difference in the clinicopathologic features between the two groups (*p* > 0.05, Chi-square test). The median follow-up time of the 317 patients was 59 months. Our cohort paralleled our earlier findings; we found that the survival times were significantly shorter in MP-OC than in P-OC (*p<* 0.05, Kaplan–Meier survival analysis) (**Figure 1C**). The median survival time of patients in MP-OC was 39.79 months (95% CI, 26.27–53.30) and 66.95 months in P-OC (95% CI, 63.33–70.57). To determine the independent risk factors for Kaplan–Meier results, univariate regression analyses and multivariate Cox regression analysis were used. The results showed that history of cancer and N stage were independent risk factor factors of OC patients (**Figure 1D**).

**Table 1:** Clinicopathologic features of 317 patients for follow-up.

	MP-OC (16)	P-OC (301)	
Clinicopathological features	n (%)	
Gender			*p*>.05
Male	8 (50.0%)	203 (67.4%)	
Female	8 (50.0%)	98 (32.6%)	
Age			*p*>.05
≥55	10 (62.5%)	148 (49.2%)	
<55	6 (37.5%)	153 (50.8%)	
Location of disease			*p*>.05
Tongue	5 (31.3%)	154 (51.2%)	
Gingiva	3 (18.8%)	33 (11.0%)	
Buccal region	2 (12.5%)	43 (14.3%)	
Floor of mouth	1 (6.3%)	24 (8.0%)	
Hard palate	1 (6.3%)	11 (3.7%)	
Other sites	4 (25.0%)	36 (12.0%)	
T stage			*p*>.05
T1-2	11 (68.8%)	204 (67.8%)	
T3-4	5 (31.3%)	97 (32.2%)	
N stage			*p*>.05
N0	10 (62.5%)	213 (70.8%)	
N+	6 (37.5%)	88 (29.2%)	
Histologic type			*p*>.05
Well-differentiated	5 (31.3%)	79 (26.2%)	
Moderately differentiated	7 (43.8%)	136 (45.2%)	
Poorly differentiated	1 (6.3%)	4 (1.3%)	
UK	3 (18.8%)	82 (27.2%)	
Smoking			*p*>.05
Yes	3 (18.8%)	117 (38.9%)	
No	13 (81.3%)	184 (61.1%)	
Excessive alcohol intake			*p*>.05
Yes	2 (12.5%)	54 (17.9%)	
No	14 (87.5%)	247 (82.1%)	

There was no significant difference in clinicopathological features between MP-OC group and P-OC groups (p value > 0.05, Chi-square test).

### 3.3 General mutation characteristics of MP-OC and P-OC

To explore the mechanisms underlying the difference in prognosis, 9 MP-OC and 21 P-OC tumor samples were analyzed by WES. The clinicopathologic features of the cases are listed in **Table 2** (further details are provided in **Supplementary Table S2**).

**Table 2:** Clinicopathologic features of 30 patients for whole exome sequencing.

	MP-OC (9)	P-OC (21)
Clinicopathological features	n (%)	
Gender		
Male	3 (33.3%)	14 (66.7%)
Female	6 (66.7%)	7 (33.3%)
Age		
≥55	6 (66.7%)	9 (42.9%)
<55	3 (33.3%)	12 (57.1%)
Smoking		
Yes	0	10 (47.6%)
No	9 (100.0%)	11 (52.4%)
Excessive drinking		
Yes	1 (11.1%)	5 (23.8%)
No	8 (88.9%)	16 (76.2%)
Chewing betel nut		
Yes	0	4 (19.0%)
No	9 (100.0%)	17 (81.0%)
Family history of cancer		
Yes	0	0
No	9 (100.0%)	21 (100.0%)
Site of oral cancer		
Tongue	4 (44.4%)	14 (66.7%)
Gingiva	2 (22.2%)	2 (4.8%)
Buccal region	1 (11.1%)	4 (19.0%)
Other sites	2 (22.2%)	1(9.5%)
T stage		
T1-2	5 (55.6%)	16 (76.2%)
T3-4	4 (44.4%)	5 (23.8%)
N stage		
N0	7 (77.8%)	15 (71.4%)
N+	2 (22.2%)	6 (28.6%)
Clinical stages		
I	0	4 (19.0%)
II	4 (44.4%)	8 (38.1%)
III	2 (22.2%)	4 (19.0%)
IV	3 (33.3%)	5 (23.8%)
Pathological differentiation		
Well-differentiated	5 (55.6%)	14 (66.7%)
Moderately differentiated	3 (33.3%)	1 (4.8%)
Poorly differentiated	0	1 (4.8%)
UK	1 (11.1%)	5 (23.8%)

An average of 91.17 million reads was acquired with an average base quality of 97.45% (Q20). The ratio of high-quality clean reads ranged from 99.24% to 99.84% (**Supplementary Figure S1A**). The average ratio of on-target coverage was 84.45%. The average target depth ranged from 66.16× to 452.29×, and 90% samples reached 80×. The quality control data for each case are provided in **Supplementary Table S3**.

**Figure 2:**
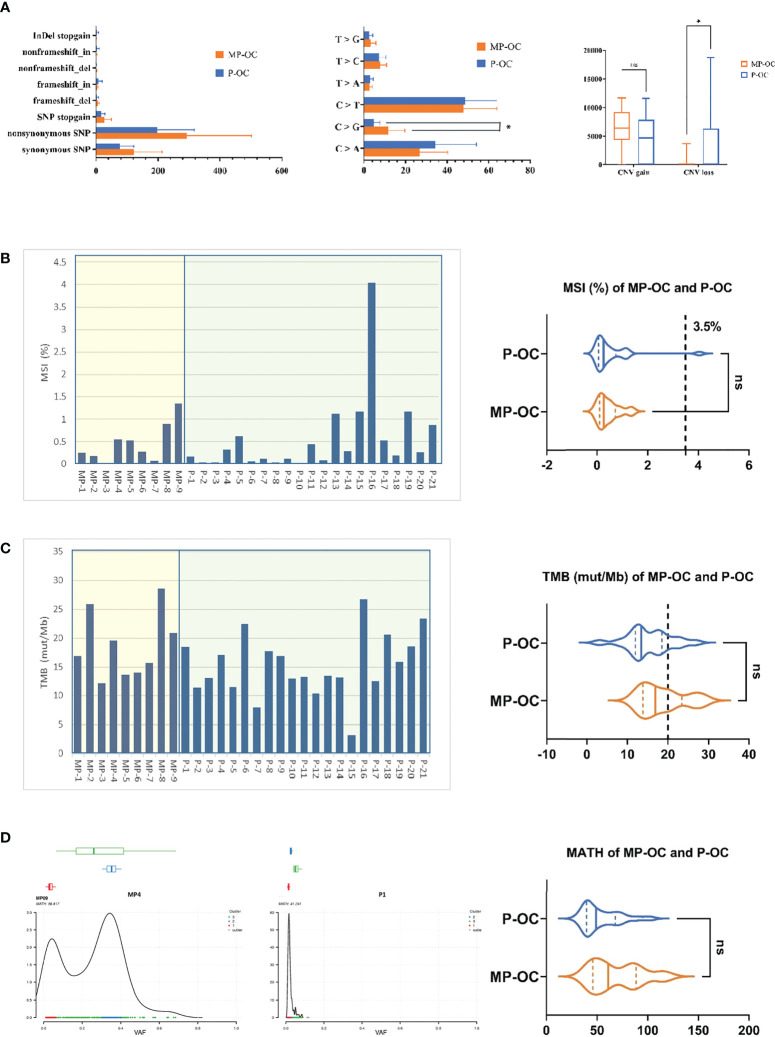
General mutation characteristics of 9 MP-OC and 21 P-OC patients. All somatic substitutions identified in this study were included, and the bar graph showed the subtype of SNP and InDel (left), proportion of base substitution types (middle), and number of copy number variation fragments (right) between MP-OC and P-OC groups, respectively. * indicates significant difference between the two groups (Mann–Whitney test, *p*< 0.05) **(A)**. TMB and MSI of 9 MP-OC patients and 21 P-OC patients. The bar chart (left) shows the details of 30 patients, and the violin diagram (right) shows the comparison of MSI and TMB between the two groups, respectively. Mann–Whitney test, ns stands for no significance, which indicates *p* > 0.05. The cutoff between TMB-H and TMB-L is 20%, and the cutoff between MSI and MSS is 3.5% **(B, C)**. The figures showing typical MATH of MP-OC (left) and P-OC (middle) patients, respectively. The right violin diagram showing the comparison of MATH between the two groups. Mann–Whitney test, ns indicates *p* > 0.05 **(D)**.

Among the 30 collected patient tumors, we observed 6,761 non-synonymous SNPs and 2,272 synonymous SNPs (**Supplementary Table S4**). The type and distribution of SNPs and InDels are listed in **Supplementary Table S4** and **Supplementary Figures S1C–F**. The SNP and InDel number of MP-OC trended higher than P-OC, although the difference was not significant. The median SNP and InDel number of MP-OC were 552 (first quartile to third quartile [Q1–Q3], 485–780) and 33 (Q1–Q3, 30–35), and that of P-OC were 410 (Q1–Q3, 356–596) and 17 (Q1–Q3, 14–50), respectively. The most common SNP types in both groups were nonsynonymous SNPs (**Figure 2A**, left; **Supplementary Table S4**). The most common base substitution in both groups was C > T. More C>G mutations were observed in MP-OC than in P-OC (*p*<0.05) (**Figure 2A**, middle; **Supplementary Table S4**).

CNV gain numbers of MP-OC trended higher than in P-OC, although the difference was not significant, and CNV loss was significantly less than in P-OC (*p*< 0.05, Mann–Whitney test). The median CNV gains of MP-OC and P-OC were 6,423 (Q1–Q3, 5,914–7,459) and 4,693 (Q1–Q3, 22–7,476), respectively. The median CNV losses of MP-OC and P-OC were 0 (Q1–Q3, 0–2) and 54 (Q1–Q3, 1–5,653), respectively (**Figure 2A**, right; **Supplementary Table S4**).

Then, we analyzed MSI, TMB, and MATH in the two groups. No significant differences in MSI, TMB, or MATH were found (**Supplementary Table S5**). MSI was 0.27% (Q1–Q3, 0.16%–0.55%) in the MP-OC group and 0.26% (Q1–Q3, 0.08%–0.62%) in the P-OC group (*p*>0.05, nonparametric Mann–Whitney test) (**Figure 2B**). Taking 3.5% as the cutoff between MSI and microsatellite stability (MSS), only one patient exceeded the threshold. TMB was 16.93% (Q1–Q3, 13.84%–23.43%) in the MP-OC group and 13.47% (Q1–Q3, 11.99%–18.55%) in the P-OC group (*p* > 0.05, nonparametric Mann–Whitney test) (**Figure 2C**). The MATH of MP was 61.12 (Q1–Q3, 45.93–88.64) and 49.22 (Q1–Q3, 39.70–68.16) in P-OC (*p* > 0.05, nonparametric Mann–Whitney test) (**Figure 2D**; **Supplementary Table S5**). TMB and MATH trended higher in the MP-OC than in the P-OC group.

### 3.4 MP-OC and P-OC have different mutational signatures

**Figure 3:**
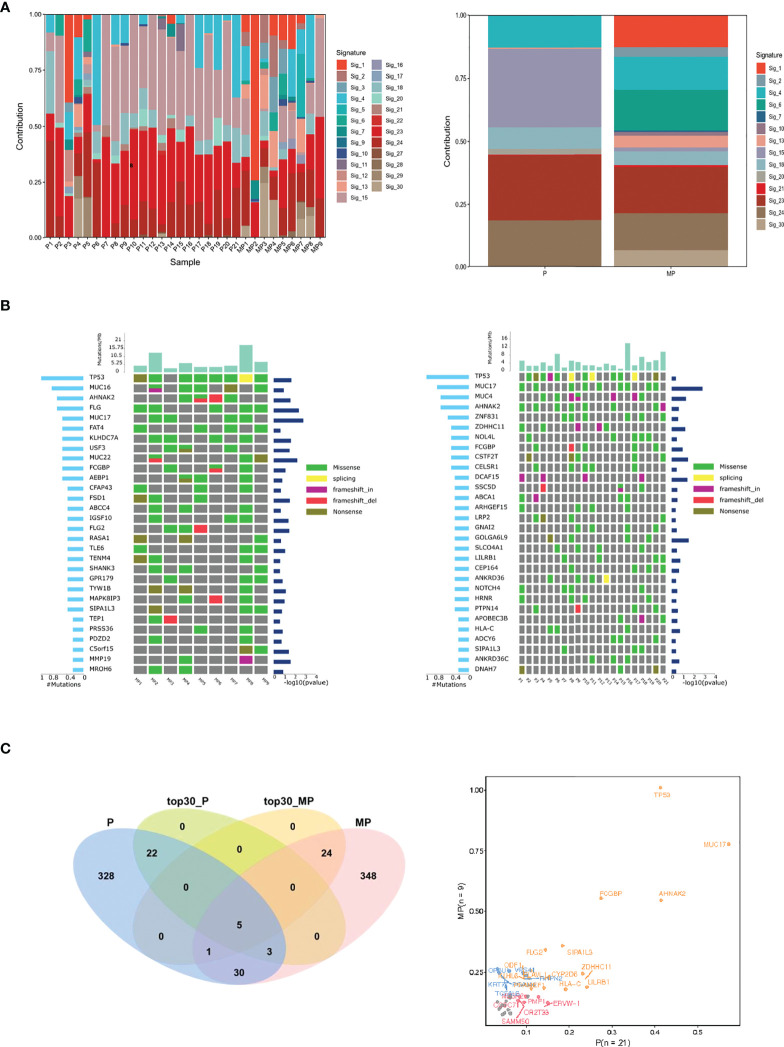
Mutational signature, frequent mutated genes of MP-OC and P-OC patients. Decomposition of mutational signatures; all somatic substitutions identified in this study were included to decipher mutational signatures. The bar plots show the details of 30 patients (left), their relative contribution, and differences between the two groups (right) **(A)**. The landscape of frequent mutated genes in MP-OC (left) and P-OC (right) groups. The middle panel shows the somatic mutations by patient (column) and by gene (row). The histogram at the top shows the number of mutations accumulated in each individual sample, and mutation types are marked with different colors **(B)**. Venn diagram showing the differences of significantly mutated genes. The right figure lists the genes with significant differences between the two groups in different colors **(C)**.

Firstly, we analyzed the mutational signatures of the two groups. Based on the catalogue of somatic mutations in cancer (COSMIC) (20), there were 14 mutational signatures in the MP-OC group and 8 mutational signatures in P-OC. The patterns of mutational signatures were more complex in MP-OC than P-OC. The somatic mutation types of the MP-OC and P-OC groups are shown in **Supplementary Figure S2**. Signatures 4, 13, 15, 18, 20, 21, 23, and 24 were shared by the two groups, while signatures 1, 2, 6, 7, 10, and 30 were unique to the MP-OC group (**Figure 3A**; **Supplementary Table S6**).

Then, we focused on the mutated genes (**Supplementary Table S6**). The spectra of mutated genes differed between the two groups. In the MP-OC group, *TP53* was the most frequently mutated gene, followed by *MUC16*, *AHNAK2*, and *FLG*. In the P-OC group, the most frequent mutation was also *TP53*, followed by *MUC17*, *MUC4, AHNAK2*, and *TTN* (**Figure 3B**). The two groups only shared 5 of the top 30 most frequently mutated genes. Only one SMG, MUC17, was observed in the P-OC group (**Figure 3C**).

**Figure 4:**
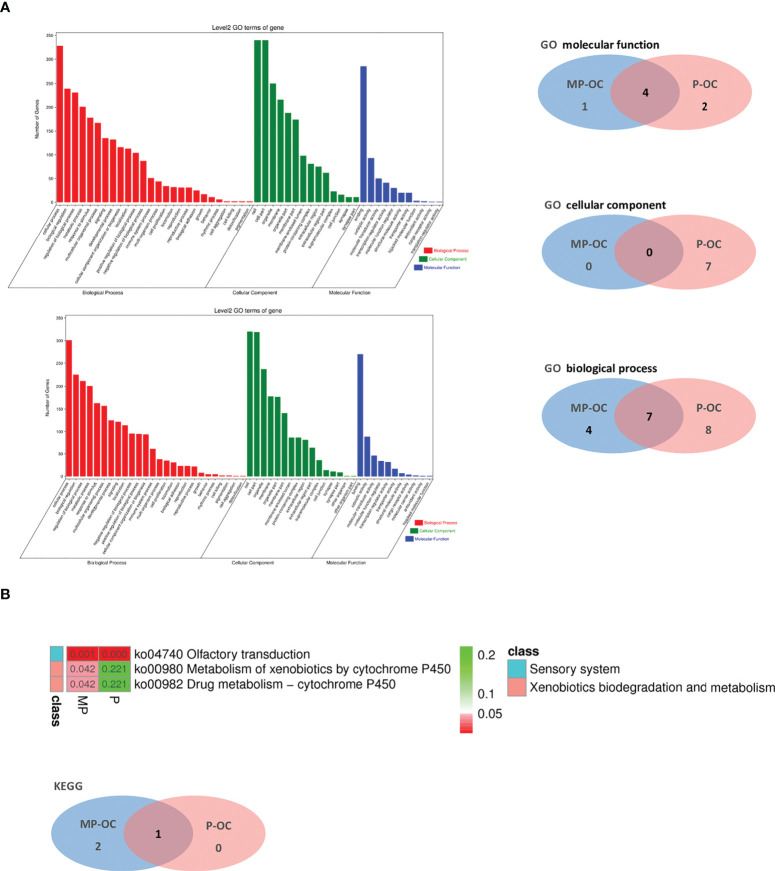
Pathway analysis of MP-OC and P-OC patients. Histogram showing the difference of GO between the MP-OC (upper) and P-OC (lower) group. Venn diagram showing the differences of molecular function, cellular component, and biological process in GO, respectively **(A)**. Lists of the significant KEGG pathways in the two groups. Wayne diagram showing the differences between the two groups **(B)**.

Lastly, we conducted pathways analysis using GO and KEGG with the mutated genes in MP-OC and P-OC. The two groups shared some of the pathways. In the cellular component (CC), molecular function (MF), and biological process (BP) parts of GO pathways, there were zero, one, and four pathways unique to the MP-OC group (**Figure 4A**). Among KEGG pathways, there were two pathways unique to the MP-OC group, including drug metabolism–cytochrome P450 and metabolism of xenobiotics by cytochrome P450 (**Figure 4B**; **Supplementary Table S6**).

### 3.5 Different CNV patterns in MP-OC and P-OC groups

**Figure 5:**
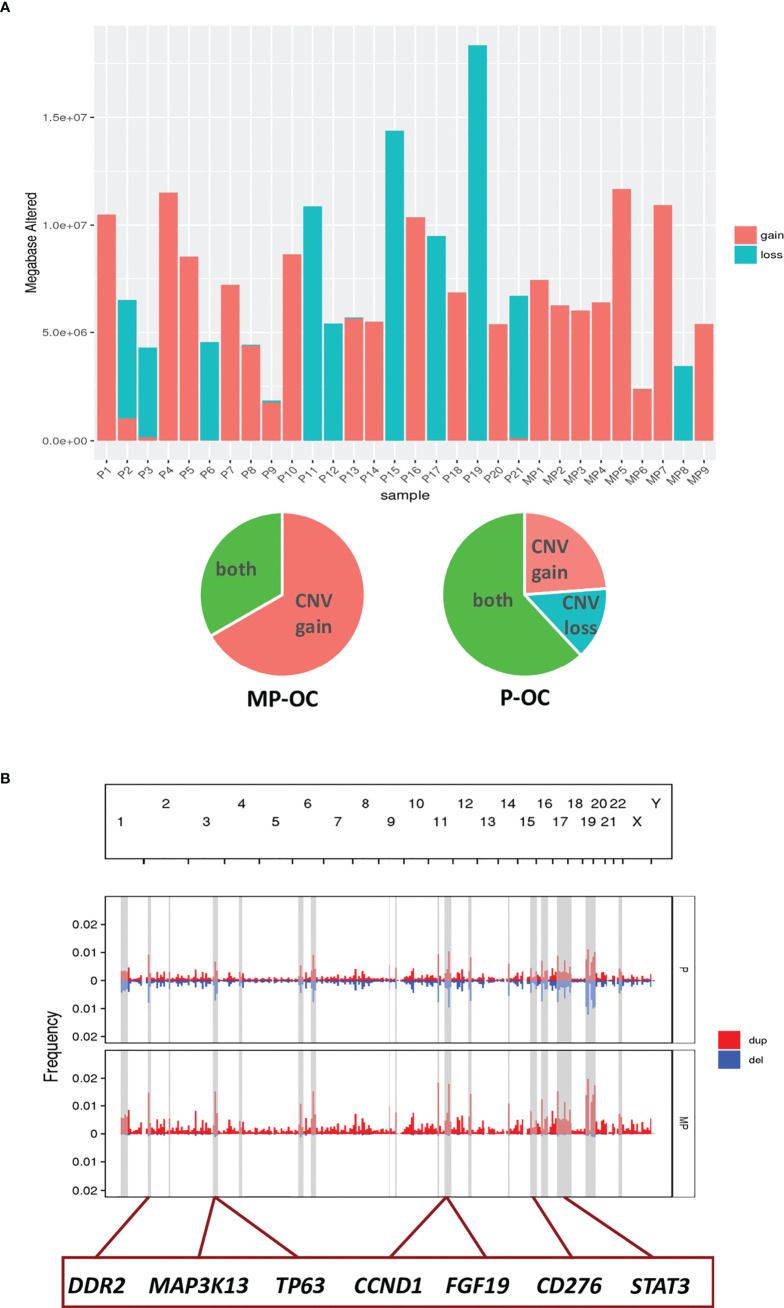
CNV in MP-OC and P-OC groups. Histogram showing CNV gains (red) and CNV loss (blue) in 9 MP-OC patients and 21 P-OC patients. Patients with CNV gains and CNV loss in the two groups were counted in the pie chart **(A)**. Composite of copy number profiles for the MP and P group, with gains in red and losses in blue. The regions that showed a difference in the frequency of copy number alterations between two groups were shaded in light gray rectangles; some genes in these regions are labeled **(B)**.

We evaluated DNA fragment changes by CNV. We observed that mainly CNV gains rather than CNV losses occurred in MP-OC. Both CNV gain and CNV loss, and mixed patterns were observed in the P-OC group (**Figure 5A**; **Supplementary Table S4**). We highlighted some of the chromosome fractions that were different between the two groups. In these fragments, there were 99 oncogenes and 89 tumor suppressor genes, while 14 genes act as a double-edged sword (**Supplementary Table S7**). We labeled some genes that may impact the pathogenesis of MP-OC (**Figure 5B**).

### 3.6 Germline mutations in MP-OC and P-OC group.

We also tried to analyze whether germline mutations were the possible cause of MP-OC. The clinicopathological features of 64 patients are shown in **Supplementary Table S2**. In our preliminary study, we found no significant differences between the two groups (*p* > 0.05, nonparametric Mann–Whitney test) (**Table 3**; **Supplementary Table S8**).

**Table 3:** Germline mutations in MP-OC and P-OC group.

	MP-OC	P-OC		MP-OC	P-OC
Symbol	n (%)		Symbol	n (%)	
*MSH6*	4 (20.0%)	0	*HBB*	0	1 (2.3%)
*SDHA*	2 (10.0%)	4 (9.1%)	*TPO*	0	1 (2.3%)
*SETBP1*	2 (10.0%)	5 (11.4%)	*EPCAM*	0	1 (2.3%)
*AGBL1*	1 (5.0%)	3 (6.8%)	*TSC2*	0	1 (2.3%)
*FLG*	1 (5.0%)	2 (4.5%)	*FANCA*	0	1 (2.3%)
*GCDH*	1 (5.0%)	0	*TP53*	0	1 (2.3%)
*SUN5*	1 (5.0%)	0	*RNF135*	0	1 (2.3%)
*CABP4*	1 (5.0%)	0	*NF1*	0	1 (2.3%)
*SLC37A4*	1 (5.0%)	0	*BRCA1*	0	1 (2.3%)
*GALNS*	1 (5.0%)	0	*RHBDF2*	0	1 (2.3%)
*ABCA4*	1 (5.0%)	0	*SERPINB7*	0	1 (2.3%)
*PRDM9*	1 (5.0%)	1 (2.3%)	*PINK1*	0	1 (2.3%)
*EVC2*	1 (5.0%)	1 (2.3%)	*HPS3*	0	1 (2.3%)
*SLCO1B1*	1 (5.0%)	1 (2.3%)	*ETFDH*	0	1 (2.3%)
*BRCA2*	1 (5.0%)	1 (2.3%)	*CYP4V2*	0	1 (2.3%)
*GJB2*	1 (5.0%)	1 (2.3%)	*SBDS*	0	1 (2.3%)
*EGF*	0	1 (2.3%)	*CD36*	0	1 (2.3%)
*F11*	0	1 (2.3%)	*CLCN1*	0	1 (2.3%)
*DNAH5*	0	1 (2.3%)	*FBP1*	0	1 (2.3%)
*PNPLA1*	0	1 (2.3%)	*TYR*	0	1 (2.3%)
*DST*	0	1 (2.3%)	*C12orf65*	0	1 (2.3%)
*EYS*	0	1 (2.3%)	*ATP7B*	0	1 (2.3%)
*SLC25A13*	0	1 (2.3%)	*GALC*	0	1 (2.3%)
*CYP7B1*	0	1 (2.3%)	*SPG11*	0	1 (2.3%)

## 4 Discussion

Firstly, we analyzed data from the SEER database. We found that in recent years, the proportion of multiple primary cancer in the total OC pool has increased. The oral cavity is one body region with a high incidence of multiple primary cancers (36). Without a medical history, it is difficult to distinguish multiple primary cancers from primary cancers in the oral cavity. These distinctions are critical, as our study shows that the prognosis of MP-OC patients is worse than P-OC patients; this finding is consistent with previous studies (8, 37, 38). However, the molecular mechanisms remain unexplored. With an increasing incidence of multiple primary OCs, there is growing importance in understanding the molecular mechanisms of poor prognosis. Therefore, we attempted to characterize the differences in the molecular mechanisms of MP-OC and P-OC by utilizing WES.

In general, mutations were more frequent in the MP-OC group. This group had a trend towards greater SNP, TMB, and MATH. TMB refers to the number of mutations per million bases in cancer. The MATH value was calculated from the MAD and the median of its mutant-allele fractions at tumor-specific mutated loci (39). MATH evaluates the genetic differences between tumor cells. Previous studies showed that TMB and MATH are not significantly different in multiple gastric cancer and multiple primary lung cancer (39, 40), and our research supports these findings. However, the situation may be more complicated in MP-OC than in P-OC. A larger sample size is essential to confirm and refine these conclusions. In addition, we also compared the MSI of the two groups. No significant differences or tendencies were found. MSI refers to short and tandem repeat base sequences that have errors in the replication process, usually arising from DNA mismatch repair defects (dMMR). Previous studies found that colorectal cancer and head and neck cancer (HNC) with MSI-H show a higher risk of MPC (41, 42). Multiple primary gastrointestinal cancer exhibits frequent MSI (43, 44). MSI-H is not common in OC. A study across 39 cancer types showed an incidence of only 0.78% in head and neck squamous cell carcinoma (45). Our research shows similar results in OC.

Mutational signatures are different combinations of mutation types, often originating from different mutational processes (46). The mutation types of MP-OC and P-OC groups are shown in Figure S2. In our study, the mutational signatures of the MP-OC group were more abundant than in the P-OC group, which might result from more complicated pathogenic factors. Signature 1 was closely related to age. The age at diagnosis of patients with MPCs is older than that of patients with the same primary cancer (47, 48). Accumulated mutations accompanied by aging might play an important role in the occurrence of MPCs. Signatures 2 and 13 were related to the activity of the AID/APOBEC cytidine deaminase family. The APOBEC family has been shown to induce tumor mutations by aberrant DNA editing mechanisms (49). Infection with high-risk HPV, which is one of the pathogenic factors in the oral and maxillofacial regions, leads to increased APOBEC family activity (50–52).

We also found that the mutation spectrum between the two groups was significantly different. Among the top 30 frequently mutated genes, only 5 overlapped between groups. Similar results were found in our GO and KEGG pathways analysis. We carefully reviewed the specific KEGG pathways peculiar to MP-OC, in which the cytochrome P450 pathway is involved. Various studies have shown the significance of CYP 450 polymorphism in the susceptibility to cancer. In hormone-related cancers such as breast cancer (53) and prostate cancer (54), CYP 450, which is involved in steroid hormone metabolism, affects cancer susceptibility. Studies have shown that patients carrying a mutation of the CYP1A1/2, CYP2D6, and CYP2E1 have an increased risk of head and neck squamous cell carcinoma, esophageal carcinoma, and lung carcinoma, arising from a deficiency in metabolizing carcinogens to their inactive derivatives (55, 56); alcohol and nicotine are common carcinogens. The metabolism of cytochrome P450 plays an important role in the production of ROS and oxidative stress (57). Mutation of the cytochrome P450 pathway may play an important role in the development of MPC. On the contrary, cigarette extracts have been shown to directly upregulate the expression of CYP 450 (58). OSCC patients who use tobacco also have some relevant characteristics, such as higher C>G transversion mutations (59). In our study, no smoker or betel nut chewer was enrolled into the MP-OC group for sequencing, which might have biased our results. Samples involving MPC smokers or nut-chewing patients will be collected in our future studies.

CNV refers to a form of genomic structural change that results in abnormal gene copy numbers (46). It is an important way to regulate the expression and function of oncogenes and tumor suppressor genes, which lead to the evolution and progression of cancer (46, 60–63). We found different CNV patterns between the two groups. The main CNV pattern of MP-OC patients was gain. Cancer patients with CNV gain have a poorer prognosis (64), which might be one of the reasons why MP-OC patients have a poorer prognosis.

The causes of MPCs include accumulation of mutations accompanied by aging, field cancerization caused by physical and chemical factors, virus infection including HPV, iatrogenic effects of radiotherapy and chemotherapy, and germline mutations (41, 65–67). We preliminarily investigated germline mutations as a potential cause. Germline mutation refers to a congenital gene mutation in germ cells and leads to a higher risk of canceration of somatic cells. However, we found no significant differences in germline mutations between MP-OC and P-OC.

## 5 Conclusions

The biological behavior and molecular mechanism underlying MPC were different from primary cancers. MP-OC patients had a worse prognosis. By WES and comparative analysis of 9 MP-OC patients and 21 P-OC patients, our study described the differences in somatic point and fragment mutations between the two groups. TMB and MATH in the MP-OC group trended higher than in P-OC. The MP-OC group had more complex mutation signatures, suggesting that age-related factors and the AID/APOBEC pathway play different roles in MP-OC versus P-OC. The mutated genes of MP-OC were enriched in the *cytochrome P450* pathway of KEGG. Amplification is the main mode of CNV in MP-OC. Our study did not find germline mutations that play a key role in the pathogenesis of MP-OC. The mechanisms underlying MPC remain appealing but unresolved. There are several limitations in our preliminary study. Smoking and nut-chewing patients were not involved in the sequencing study. A larger cohort that includes smoking and nut-chewing patients will be essential for further studies. Samples should be carefully stratified and analyzed according to the different potential causes, including aging, field cancerization, radiation, and germline mutations. Moreover, WES alone is unable to resolve molecular mechanisms. Multi-omics studies incorporating analyses of the transcriptome and proteome will need to be incorporated to tease out the biological effects of specific mutations. *In vivo* and *in vitro* experiments need to be performed to confirm the specific potential driving genes and pathogenic pathways.

## Data availability statement

The raw sequence data reported in this paper have been deposited in the Genome Sequence Archive in National Genomics Data Center, China National Center for Bioinformation/Beijing Institute of Genomics, Chinese Academy of Sciences (GSA-Human: HRA002206) and are publicly accessible at https://ngdc.cncb.ac.cn/gsa-human/s/ov8sC0i6.

## Ethics statement

The studies involving human participants were reviewed and approved by Ethics Committee of Hospital of Stomatology, Sun Yat-sen University. The patients/participants provided their written informed consent to participate in this study. Written informed consent was obtained from the individual(s) for the publication of any data included in this article.

## Author contributions

KL and JG drafted the article and reviewed the submitted version of the manuscript. QZ worked on the acquisition of data. KL, JG, QZ, LY, and XM worked on the analysis and interpretation of data. LY, XM, JC, GL, and YL critically revised the article and worked on the statistical analysis. GL and YL are responsible for administrative/technical/material support and study supervision. KL, JG, and YL worked on conception and design. YL and GL approved the final version of the manuscript on behalf of all authors. All authors contributed to the article and approved the submitted version.

## Funding

This work was supported by the National Natural Science Foundation of China (Nos. 81902768, 82072995, and 81972544).

## Acknowledgments

We appreciate all the patients and investigators involved in this study. We are grateful to Guangzhou Gene Denovo Honour Biotechnology Co., Ltd for assisting in sequencing and bioinformatics analysis.

## Conflict of interest

The authors declare that the research was conducted in the absence of any commercial or financial relationships that could be construed as a potential conflict of interest.

## Publisher’s note

All claims expressed in this article are solely those of the authors and do not necessarily represent those of their affiliated organizations, or those of the publisher, the editors and the reviewers. Any product that may be evaluated in this article, or claim that may be made by its manufacturer, is not guaranteed or endorsed by the publisher.

## References

[1] Vogt A Schmid S Heinimann K Frick H Herrmann C Cerny T. Multiple primary tumours: challenges and approaches, a review. ESMO Open (2017) 2(2):e000172. doi: 10.1136/esmoopen-2017-000172 28761745 PMC5519797

[2] Billroth T. Die allgemeine chirurgische Pathologie and Therapie. In Reimer G ed. 51 Vorlesungen - Ein Handbuch für Studierende and Ärzter, ed 14. Berlin, Reimer (1889).

[3] Warren S. Multiple primary malignant tumors. a survey of the literature and a statistical study. Am J Cancer (1932) 16:1358–414.

[4] Copur MS Manapuram S. Multiple primary tumors over a lifetime. Oncol (Williston Park) (2019) 33(7):280-283. 31365752

[5] Sung H Ferlay J Siegel RL Laversanne M Soerjomataram I Jemal A. Global cancer statistics 2020: GLOBOCAN estimates of incidence and mortality worldwide for 36 cancers in 185 countries. CA: Cancer J Clin (2021) 71(3):209–49. doi: 10.3322/caac.21660 33538338

[6] Barclay ME Lyratzopoulos G Walter FM Jefferies S Peake MD Rintoul RC. Incidence of second and higher order smoking-related primary cancers following lung cancer: A population-based cohort study. Thorax (2019) 74(5):466–72. doi: 10.1136/thoraxjnl-2018-212456 PMC6475108 30777897

[7] Feller A Matthes KL Bordoni A Bouchardy C Bulliard JL Herrmann C. The relative risk of second primary cancers in Switzerland: A population-based retrospective cohort study. BMC Canc (2020) 20(1):51. doi: 10.1186/s12885-020-6584-2 PMC6974968 31964352

[8] Ko KH Huang HK Chen YI Chang H Tsai WC Huang TW. Surgical outcomes of second primary lung cancer after the extrapulmonary malignancy. J Cancer Res Clin Oncol (2020) 146(12):3323–32. doi: 10.1007/s00432-020-03310-x PMC11804703 32632580

[9] Li QW Zhu YJ Zhang WW Yang H Liang Y Hu YH. Chemoradiotherapy for synchronous multiple primary cancers with esophageal squamous cell carcinoma: A case-control study. J Canc (2017) 8(4):563–9. doi: 10.7150/jca.17408 PMC5370500 28367236

[10] Bertolini F Trudu L Banchelli F Schipilliti F Napolitano M Alberici MP. Second primary tumors in head and neck cancer patients: The importance of a "tailored" surveillance. Oral Dis (2021) 27(6):1412–20. doi: 10.1111/odi.13681 33051941

[11] Dimaras H Corson TW Cobrinik D White A Zhao J Munier FL. Retinoblastoma. Nat Rev Dis Primers (2015) 1:15021. doi: 10.1038/nrdp.2015.21 27189421 PMC5744255

[12] Zhou C Chen S Xu F Wei J Zhou X Wu Z. Estimating tumor mutational burden across multiple cancer types using whole-exome sequencing. Ann Transl Med (2021) 9(18):1437. doi: 10.21037/atm-21-4227 34733989 PMC8506705

[13] Ebili HO Agboola AO Rakha E. MSI-WES: A simple approach for microsatellite instability testing using whole exome sequencing. Future Oncol (2021) 17(27):3595–606. doi: 10.2217/fon-2021-0132 34291669

[14] Mao W Zhang Z Huang X Fan J Geng J. Marital status and survival in patients with penile Cancer. J Canc (2019) 10(12):2661–9. doi: 10.7150/jca.32037 PMC6584924 31258774

[15] Hankey BF Ries LA Edwards BK. The surveillance, epidemiology, and end results program: A national resource. Cancer Epidemiol Prev Biomark (1999) 8(12):1117–21. 10613347

[16] Chen S Zhou Y Chen Y Gu J. Fastp: An ultra-fast all-in-one FASTQ preprocessor. Bioinformatics (2018) 34(17):i884–i90. doi: 10.1093/bioinformatics/bty560 PMC6129281 30423086

[17] Li H Durbin R. Fast and accurate short read alignment with burrows-wheeler transform. Bioinformatics (2009) 25(14):1754–60. doi: 10.1093/bioinformatics/btp324 PMC2705234 19451168

[18] Cibulskis K Lawrence MS Carter SL Sivachenko A Jaffe D Sougnez C. Sensitive detection of somatic point mutations in impure and heterogeneous cancer samples. Nat Biotechnol (2013) 31(3):213–9. doi: 10.1038/nbt.2514 PMC3833702 23396013

[19] Alexandrov LB Nik-Zainal S Wedge DC Campbell PJ Stratton MR. Deciphering signatures of mutational processes operative in human cancer. Cell Rep (2013) 3(1):246–59. doi: 10.1016/j.celrep.2012.12.008 PMC3588146 23318258

[20] Alexandrov LB Nik-Zainal S Wedge DC Aparicio SA Behjati S Biankin AV. Signatures of mutational processes in human cancer. Nature (2013) 500(7463):415–21. doi: 10.1038/nature12477 PMC3776390 23945592

[21] Dees ND Zhang Q Kandoth C Wendl MC Schierding W Koboldt DC. MuSiC: identifying mutational significance in cancer genomes. Genome Res (2012) 22(8):1589–98. doi: 10.1101/gr.134635.111 PMC3409272 22759861

[22] Kanehisa M Goto S. KEGG: kyoto encyclopedia of genes and genomes. Nucleic Acids Res (2000) 28(1):27–30. doi: 10.1093/nar/28.1.27 10592173 PMC102409

[23] Ashburner M Ball CA Blake JA Botstein D Butler H Cherry JM. Gene ontology: tool for the unification of biology. the gene ontology consortium. Nat Genet (2000) 25(1):25–9. doi: 10.1038/75556 PMC3037419 10802651

[24] Zhang L Li B Peng Y Wu F Li Q Lin Z. The prognostic value of TMB and the relationship between TMB and immune infiltration in head and neck squamous cell carcinoma: A gene expression-based study. Oral Oncol (2020) 110:104943. doi: 10.1016/j.oraloncology.2020.104943 32919362

[25] Niu B Ye K Zhang Q Lu C Xie M McLellan MD. MSIsensor: microsatellite instability detection using paired tumor-normal sequence data. Bioinformatics (2014) 30(7):1015–6. doi: 10.1093/bioinformatics/btt755 PMC3967115 24371154

[26] Mroz EA Rocco JW. MATH, a novel measure of intratumor genetic heterogeneity, is high in poor-outcome classes of head and neck squamous cell carcinoma. Oral Oncol (2013) 49(3):211–5. doi: 10.1016/j.oraloncology.2012.09.007 PMC3570658 23079694

[27] Wang K Li M Hakonarson H. ANNOVAR: functional annotation of genetic variants from high-throughput sequencing data. Nucleic Acids Res (2010) 38(16):e164. doi: 10.1093/nar/gkq603 20601685 PMC2938201

[28] Mermel CH Schumacher SE Hill B Meyerson ML Beroukhim R Getz G. GISTIC2.0 facilitates sensitive and confident localization of the targets of focal somatic copy-number alteration in human cancers. Genome Biol (2011) 12(4):R41. doi: 10.1186/gb-2011-12-4-r41 21527027 PMC3218867

[29] Huang KL Mashl RJ Wu Y Ritter DI Wang J Oh C. Pathogenic germline variants in 10,389 adult cancers. Cell (2018) 173(2):355–70 e14. doi: 10.1016/j.cell.2018.03.039 29625052 PMC5949147

[30] Koboldt DC Zhang Q Larson DE Shen D McLellan MD Lin L. VarScan 2: somatic mutation and copy number alteration discovery in cancer by exome sequencing. Genome Res (2012) 22(3):568–76. doi: 10.1101/gr.129684.111 PMC3290792 22300766

[31] McKenna A Hanna M Banks E Sivachenko A Cibulskis K Kernytsky A. The genome analysis toolkit: A MapReduce framework for analyzing next-generation DNA sequencing data. Genome Res (2010) 20(9):1297–303. doi: 10.1101/gr.107524.110 PMC2928508 20644199

[32] Ye K Schulz MH Long Q Apweiler R Ning Z. Pindel: A pattern growth approach to detect break points of large deletions and medium sized insertions from paired-end short reads. Bioinformatics (2009) 25(21):2865–71. doi: 10.1093/bioinformatics/btp394 PMC2781750 19561018

[33] Kumar P Henikoff S Ng PC. Predicting the effects of coding non-synonymous variants on protein function using the SIFT algorithm. Nat Protoc (2009) 4(7):1073–81. doi: 10.1038/nprot.2009.86 19561590

[34] Adzhubei I Jordan DM Sunyaev SR. Predicting functional effect of human missense mutations using PolyPhen-2. Curr Protoc Hum Genet (2013) Chapter 7:Unit7 20. doi: 10.1002/0471142905.hg0720s76 PMC4480630 23315928

[35] Niu B Scott AD Sengupta S Bailey MH Batra P Ning J. Protein-structure-guided discovery of functional mutations across 19 cancer types. Nat Genet (2016) 48(8):827–37. doi: 10.1038/ng.3586 PMC5315576 27294619

[36] Tanjak P Suktitipat B Vorasan N Juengwiwattanakitti P Thiengtrong B Songjang C. Risks and cancer associations of metachronous and synchronous multiple primary cancers: A 25-year retrospective study. BMC Canc (2021) 21(1):1045. doi: 10.1186/s12885-021-08766-9 PMC8461969 34556087

[37] Baba Y Yoshida N Kinoshita K Iwatsuki M Yamashita YI Chikamoto A. Clinical and prognostic features of patients with esophageal cancer and multiple primary cancers: A retrospective single-institution study. Ann Surg (2018) 267(3):478–83. doi: 10.1097/SLA.0000000000002118 28151796

[38] Wang H Hou J Zhang G Zhang M Li P Yan X. Clinical characteristics and prognostic analysis of multiple primary malignant neoplasms in patients with lung cancer. Cancer Gene Ther (2019) 26(11-12):419–26. doi: 10.1038/s41417-019-0084-z 30700800

[39] Yang R Li P Wang D Wang L Yin J Yu B. Genetic and immune characteristics of multiple primary lung cancers and lung metastases. Thorac Canc (2021) 12(19):2544–50. doi: 10.1111/1759-7714.14134 PMC8487821 34510768

[40] Wang A Li Z Wang M Jia S Chen J Ji K. Molecular characteristics of synchronous multiple gastric cancer. Theranostics (2020) 10(12):5489–500. doi: 10.7150/thno.42814 PMC7196298 32373223

[41] Kong P Wu R Lan Y He W Yang C Yin C. Association between mismatch-repair genetic variation and the risk of multiple primary cancers: A meta-analysis. J Canc (2017) 8(16):3296–308. doi: 10.7150/jca.19810 PMC5665047 29158803

[42] Deganello A Gitti G Mannelli G Meccariello G Gallo O. Risk factors for multiple malignancies in the head and neck. Otolaryngol Head Neck Surg (2013) 149(1):105–11. doi: 10.1177/0194599813484273 23535708

[43] Ohtani H Yashiro M Onoda N Nishioka N Kato Y Yamamoto S. Synchronous multiple primary gastrointestinal cancer exhibits frequent microsatellite instability. Int J Canc (2000) 86(5):678–83. doi: 10.1002/(SICI)1097-0215(20000601)86:5<678::AID-IJC12>3.0.CO;2-O 10797290

[44] Yamashita K Arimura Y Kurokawa S Itoh F Endo T Hirata K. Microsatellite instability in patients with multiple primary cancers of the gastrointestinal tract. Gut (2000) 46(6):790–4. doi: 10.1136/gut.46.6.790 PMC1756432 10807889

[45] Bonneville R Krook MA Kautto EA Miya J Wing MR Chen HZ. Landscape of microsatellite instability across 39 cancer types. JCO Precis Oncol (2017) 2017:1–15. doi: 10.1200/PO.17.00073 PMC5972025 29850653

[46] Liang L Fang JY Xu J. Gastric cancer and gene copy number variation: emerging cancer drivers for targeted therapy. Oncogene (2016) 35(12):1475–82. doi: 10.1038/onc.2015.209 26073079

[47] Mochizuki Y Harada H Ikuta M Shimamoto H Tomioka H Tanaka K. Clinical characteristics of multiple primary carcinomas of the oral cavity. Oral Oncol (2015) 51(2):182–9. doi: 10.1016/j.oraloncology.2014.11.013 25498922

[48] Lin JD Lin KJ Chao TC Hseuh C Tsang NM Huang BY. Clinical presentations of thyroid cancer patients with multiple primary cancers. J Endocrinol Invest (2011) 34(11):824–30. doi: 10.3275/7747 21613811

[49] Revathidevi S Murugan AK Nakaoka H Inoue I Munirajan AK. APOBEC: A molecular driver in cervical cancer pathogenesis. Cancer Lett (2021) 496:104–16. doi: 10.1016/j.canlet.2020.10.004 PMC7539941 33038491

[50] Riva G Albano C Gugliesi F Pasquero S Pacheco SFC Pecorari G. HPV meets APOBEC: New players in head and neck cancer. Int J Mol Sci (2021) 22(3):14. doi: 10.3390/ijms22031402 PMC7866819 33573337

[51] Gillison ML Akagi K Xiao W Jiang B Pickard RKL Li J. Human papillomavirus and the landscape of secondary genetic alterations in oral cancers. Genome Res (2019) 29(1):1–17. doi: 10.1101/gr.241141.118 PMC6314162 30563911

[52] Faden DL Kuhs KAL Lin M Langenbucher A Pinheiro M Yeager M. APOBEC mutagenesis is concordant between tumor and viral genomes in HPV-positive head and neck squamous cell carcinoma. Viruses (2021) 13(8):10. doi: 10.3390/v13081666 PMC8402723 34452530

[53] Ibrahem SQ Ahmed HQ Amin KM. Genetic variations in cytochrome P450 1A1 and 1B1 genes in a cohort of patients from Iraq diagnosed with breast cancer. Breast Cancer (Auckl) (2021) 15:11782234211050727. doi: 10.1177/11782234211050727 34671182 PMC8521753

[54] Kmetova Sivonova M Jurecekova J Tatarkova Z Kaplan P Lichardusova L Hatok J. The role of CYP17A1 in prostate cancer development: structure, function, mechanism of action, genetic variations and its inhibition. Gen Physiol Biophys (2017) 36(5):487–99. doi: 10.4149/gpb_2017024 29372682

[55] Alamgir MM Jamal Q Mirza T. Genetic profiles of different ethnicities living in Karachi as regards to tobacco-metabolising enzyme systems and the risk of oral cancer. J Pak Med Assoc (2022) 72(6):1092–6. doi: 10.47391/JPMA.3053 35751315

[56] Mittal B Tulsyan S Kumar S Mittal RD Agarwal G. Cytochrome P450 in cancer susceptibility and treatment. Adv Clin Chem (2015) 71:77–139. doi: 10.1016/bs.acc.2015.06.003 26411412

[57] Guo Z Johnson V Barrera J Porras M Hinojosa D Hernandez I. Targeting cytochrome P450-dependent cancer cell mitochondria: cancer associated CYPs and where to find them. Cancer Metastasis Rev (2018) 37(2-3):409–23. doi: 10.1007/s10555-018-9749-6 30066055

[58] Richter GM Kruppa J Munz M Wiehe R Hasler R Franke A. A combined epigenome- and transcriptome-wide association study of the oral masticatory mucosa assigns CYP1B1 a central role for epithelial health in smokers. Clin Epigenet (2019) 11(1):105. doi: 10.1186/s13148-019-0697-y PMC6647091 31331382

[59] India Project Team of the International Cancer Genome C. Mutational landscape of gingivo-buccal oral squamous cell carcinoma reveals new recurrently-mutated genes and molecular subgroups. Nat Commun (2013) 4:2873. doi: 10.1038/ncomms3873 24292195 PMC3863896

[60] Lynch MR Kuhn HG Carey RJ. Chronic haloperidol-amphetamine interactions and mesolimbic dopamine. Neuropsychobiology (1988) 19(2):97–103. doi: 10.1159/000118442 3226530

[61] Arlt MF Rajendran S Birkeland SR Wilson TE Glover TW. Copy number variants are produced in response to low-dose ionizing radiation in cultured cells. Environ Mol Mutagen (2014) 55(2):103–13. doi: 10.1002/em.21840 PMC4086151 24327335

[62] Hong J Gresham D. Molecular specificity, convergence and constraint shape adaptive evolution in nutrient-poor environments. PloS Genet (2014) 10(1):e1004041. doi: 10.1371/journal.pgen.1004041 24415948 PMC3886903

[63] Todd RT Selmecki A. Expandable and reversible copy number amplification drives rapid adaptation to antifungal drugs. Elife (2020) 9:33. doi: 10.7554/eLife.58349 PMC7371428 32687060

[64] Liu L Bai X Wang J Tang X-R Wu D-H Du S-S. Combination of TMB and CNA stratifies prognostic and predictive responses to immunotherapy across metastatic cancer. Clin Cancer Res (2019) 25(24):7413–23. doi: 10.1158/1078-0432.CCR-19-0558 31515453

[65] Curtius K Wright NA Graham TA. An evolutionary perspective on field cancerization. Nat Rev Canc (2018) 18(1):19–32. doi: 10.1038/nrc.2017.102 29217838

[66] Wright JD St Clair CM Deutsch I Burke WM Gorrochurn P Sun X. Pelvic radiotherapy and the risk of secondary leukemia and multiple myeloma. Cancer (2010) 116(10):2486–92. doi: 10.1002/cncr.25067 20209618

[67] Neumann F Jegu J Mougin C Pretet JL Guizard AV Lapotre-Ledoux B. Risk of second primary cancer after a first potentially-human papillomavirus-related cancer: A population-based study. Prev Med (2016) 90:52–8. doi: 10.1016/j.ypmed.2016.06.041 27370167

