# Supplementary material for: Preliminary study on the molecular features of mutation in multiple primary oral cancer by whole exome sequencing
Source: Front Oncol. 2022 Oct 20;12:971546. doi: 10.3389/fonc.2022.971546 (PMC9632273; doi:10.3389/fonc.2022.971546)
Supplement: Supplementary file 1 [file DataSheet_1.zip › Supplementary Figures/Supplementary Figure Captions.docx]

***Supplementary Figure Captions***

**Figure S1** The ratio of high-quality clean reads of our data**（A）**.Types and distriction of Somatic SNP**(B)**.Types and distriction of Indel**(C)**.

**Figure S2** The 96 somatic mutation types of MP-OC and P-OC group.
