# Supplementary figures and images for: Preliminary study on the molecular features of mutation in multiple primary oral cancer by whole exome sequencing
Source: Front Oncol. 2022 Oct 20;12:971546. doi: 10.3389/fonc.2022.971546 (PMC9632273; doi:10.3389/fonc.2022.971546)

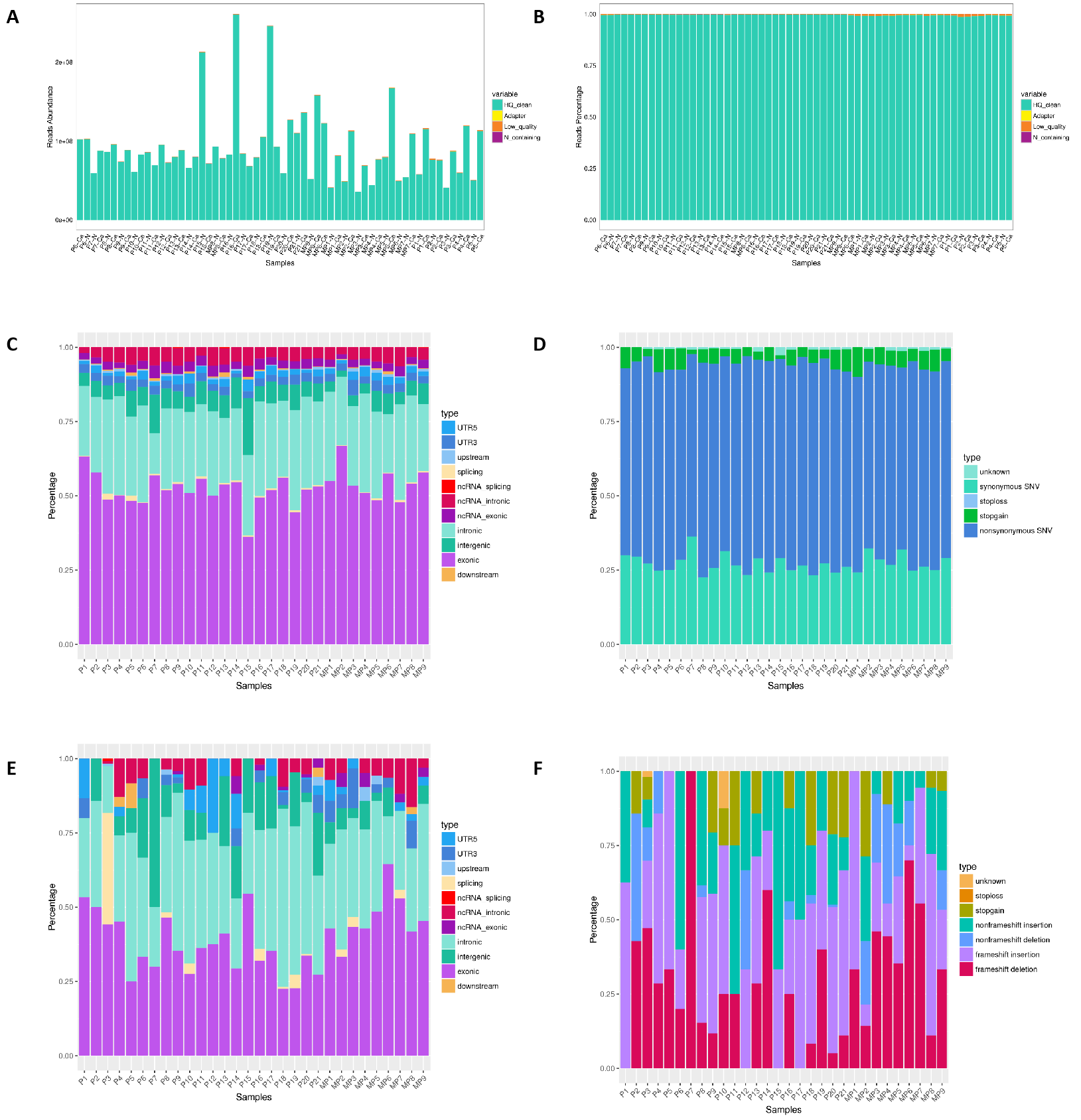

Supplement: Supplementary file 1 [file DataSheet_1.zip › Supplementary Figures/Supplementary Figure S1.tiff]

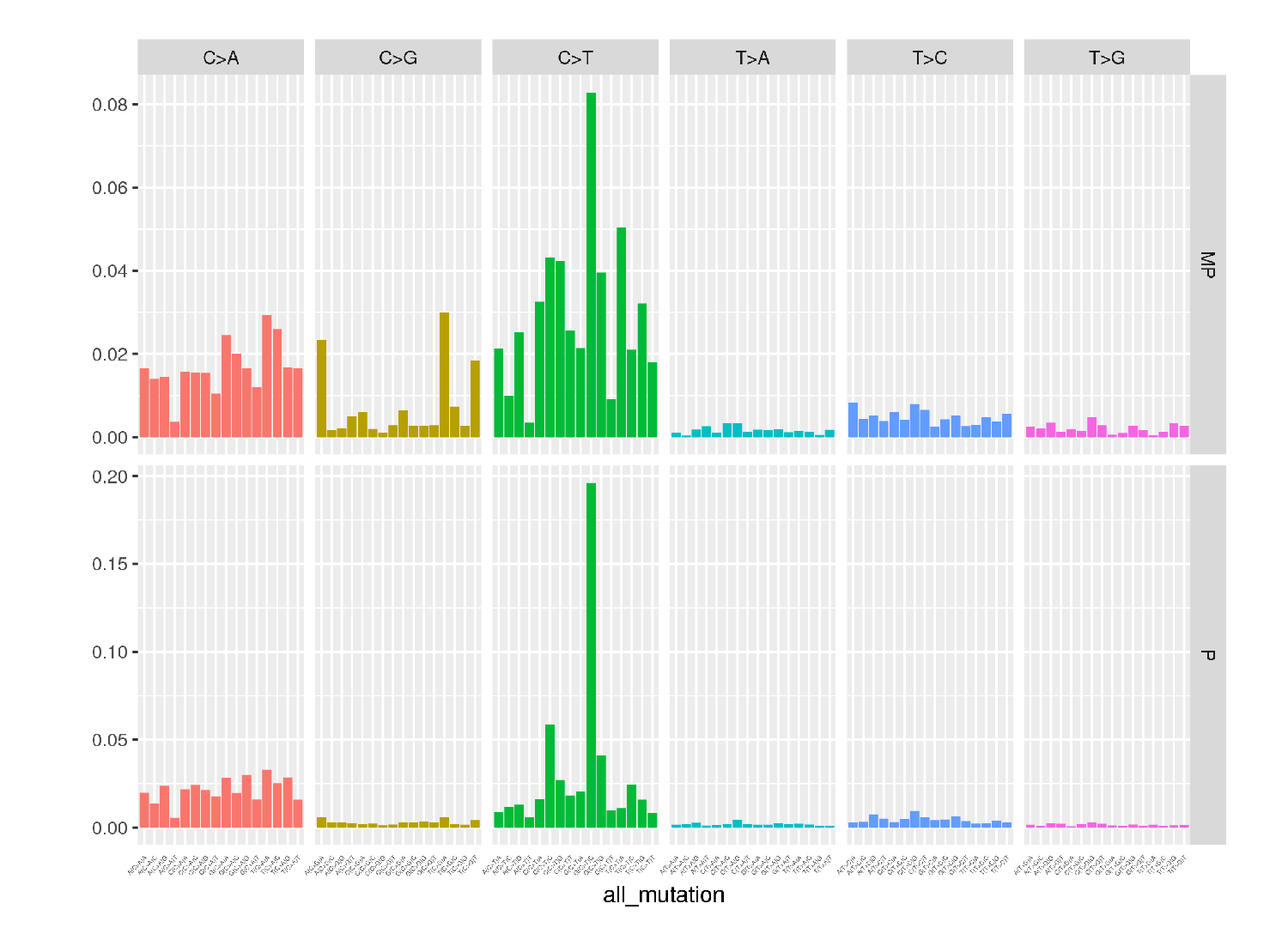

Supplement: Supplementary file 1 [file DataSheet_1.zip › Supplementary Figures/Supplementary Figure S2.TIFF]
